# The protective mechanism of SIRT1 on cartilage through regulation of LEF-1

**DOI:** 10.1186/s12891-021-04516-x

**Published:** 2021-07-27

**Authors:** Xueyu Hu, Gangning Feng, Zhiqiang Meng, Long Ma, Qunhua Jin

**Affiliations:** 1 Ningxia Medical University, The General Hospital of Ningxia Medical University, Yinchuan, Ningxia 750004 P.R. China; 2 Orthopedics Ward 3, The General Hospital of Ningxia Medical University, 804 Shengli South Street, Yinchuan, 750004 Ningxia P.R. China

**Keywords:** Osteoarthritis, Cartilage, SIRT-1, LEF-1

## Abstract

**Background:**

Osteoarthritis (OA) is a chronic degenerative disease that suppresses middle-aged and older people worldwide. Silent information regulator 1(SIRT-1) is associated with several age-related diseases, such as cardiovascular diseases, neurodegenerative diseases and tumors, etc. The protective role of SIRT-1 in bone and joint diseases has become increasingly well known.

**Objective:**

To explore the relationship between SIRT-1 and its related factors in OA.

**Methods:**

Fresh tibial plateau specimens were collected from 30 patients with knee OA who underwent total knee arthroplasty. According to the results of Safranin O Fast Green Staining, hematoxylin–eosin staining and the OARSI grade developed by the International Association for the Study of Osteoarthropathy, the specimens were divided into the mild group, moderate group and severe group, and the damage of cartilage was evaluated. SIRT-1 protein levels in cartilage samples were analyzed by immunohistochemistry. Then, take 60 8-week-old female C57BL/6 J mice and apply the Destabilization of the medial meniscus (DMM) to induce OA. Mice were randomly divided into normal group (sham), model group (model), and post-modeling drug administration group (srt), and each group was further divided into 2 weeks after modeling (2 W) and 8 weeks after modeling (8 W) according to the time after surgery. The degenerative degree of a knee joint in mouse knee cartilage samples was evaluated using Safranin O Fast Green Staining and OARSI grade. Immunohistochemical techniques assessed the protein levels of SIRT-1, β-catenin, LEF-1, MMP-13 and Collagen II in cartilage samples. The protein levels of β-catenin, LEF-1 and MMP-13 in the samples were assessed by the immunohistofluorescence technique. The mRNA expression of SIRT-1 and LEF-1 in mouse cartilage samples was evaluated by real-time quantitative polymerase chain reaction (qPCR).

**Results:**

In the human cartilage samples, according to the results of Safranin O Fast Green Staining, compared with the mild group, the moderate group and the severe group showed damage cartilage layer structure, the number of chondrocytes decreased, the cell hypertrophic, the cartilage surface discontinuous, and the OARSI grade increased. The severe group had severe cartilage injury and the highest OARSI grade. In the mice cartilage samples, according to immunohistochemical analysis, the protein levels of β-catenin, LEF-1 and MMP-13 in cartilage specimens of model 2 W and model 8 W groups were significantly increased than the sham 2 W and sham 8 W groups. The protein levels of SIRT-1 and Collagen II were significantly decreased (*P* < 0.05), the results of srt 2 W and srt 8 W groups were between the sham group and the model group. According to immunofluorescence analysis, the protein levels of β-catenin, LEF-1 and MMP-13 in model 2 W and model 8 W groups were significantly increased than sham 2 W and sham 8 W groups. The results of srt 2w and srt 8w groups were between the sham group and the model group. According to the real-time qPCR results: Compared with sham 2 W and sham 8 W groups, the mRNA expression of SIRT-1 in model 2 W and model 8 W groups was significantly decreased, while the mRNA expression of LEF-1 was significantly increased. In contrast, the results of srt 2 W and srt 8 W groups were between the sham group and the model group.

**Conclusion:**

SRT-1720, as a specific activator of SIRT-1, does increase the protein level of SIRT-1. SIRT-1 may play a protective role in cartilage by regulating the expression of LEF-1 and related inflammatory factors in OA.

## Introduction

Osteoarthritis (OA) is a chronic degenerative joint disease that affects middle and older aged people worldwide. OA is mainly caused by loss of articular cartilage, joint soft tissue remodeling, and changes in synovial inflammatory [1]. Structural changes characterize progressive damage to articular cartilage. Cartilage is mainly composed of chondrocytes and extracellular matrix. Chondrocytes account for about 1% of the total cartilage volume, and the rest of the mass is formed by the extracellular matrix [2, 3]. The extracellular matrix is rich in collagen fibers and aggrecan, which can nourish, support, and protect the cartilage cells. Various studies have shown that during the prognosis of OA, articular chondrocytes secrete pro-inflammatory cytokines (IL-1βand TNF-α) and matrix decomposing factors such as MMP-13, A Disintegrin And Metalloproteinase With Thrombospondin (ADAMTS) [4]. The increased expression of these proteins aggravates the breakdown of the extracellular matrix and significantly affects the survival of chondrocytes. Thus, leading to the occurrence and progression of OA pathological processes.

Histone deacetylase (HDAC) can specifically modify chromatin structure and regulate transcription factor activity. It catalyzes the acetyl groups from the histone’s tails and other proteins to form deacetylated substrates, acetyl groups, and a water molecule [5]. SIRT-1 is a class III HDAC, which has been proposed to inhibit OA. Specimens of subchondral osteoblast from OA patients show a significant reduction in the expression of the SIRT-1 gene compared to normal adult joint specimens [6]. In the articular cartilage of SIRT-1-deficient mice, chondrocyte apoptosis was increased while the expression of type II collagen in the extracellular matrix was significantly decreased. The expression of aggregate MMP-13 was also increased. In addition, it confirmed that the high expression of the SIRT1 gene could halt the inflammatory changes by inhibiting IL-1β in human osteoarticular chondrocytes [7]. However, the specific effect of SIRT-1 on cartilage is still not clear.

LEF-1 is a T-cell factor (TCF)/LEF family member. LEF-1 is the main downstream regulator of the Wnt signaling pathway and belongs to the high mobility group (HMG) box family [8]. LEF-1 regulates the cell cycle and cell proliferation-related genes, such as cyclin D1 and c-myc [9, 10]. Groucho /TLE can bind two domains of LEF-1 in an HDAC-dependent manner and can competitively inhibit β-catenin/LEF-1complex activity [11]. Previous studies indicated that SIRT-1 could inhibit the expression of MMP-13 in human chondrocytes by regulating LEF-1 and has a crucial chondroprotective effect [12]. However, the interaction between SIRT1 and LEF1 in the OA has not yet been fully elucidated. In this study, we conducted experiments on human and mouse knee joint specimens to study the expression relationship.

## Materials and methods

### Ethical statement

All experiments were approved by the Animal Experiment Ethics Committee of Ningxia Medical University (protocol number: 2019–066) and approved by the Medical Research Ethics Review Committee of the General Hospital of Ningxia Medical University (Proposal Number: 2020–999). All experiments were conducted under the standard principles of animal experiment ethics and comply with the Declaration of Helsinki.

### Specimen

A total of 30 patients, including 18 females and 12 males, (mean age, 68.73 ± 5.30 years; range, 58–79 years),were selected from the fresh tibial plateau tissue donated voluntarily after total knee arthroplasty for primary osteoarthritis patients admitted to the Third Department of Orthopedics of the General Hospital Of Ningxia Medical University from February to July 2020. Secondary arthritis caused by rheumatoid, trauma, and severe liver and kidney diseases were excluded.

Sixty female C57BL/6 J mice were selected, 8 weeks old (about 20 g), divided into six groups: 2 weeks after modeling (*n* = 10, model 2w), 2 weeks after modeling, srt treatment group (*n* = 10, Srt 2w), 10-week-old normal group (*n* = 10, sham 2w), 8 weeks after modeling (*n* = 10, model 8w), 8 weeks after modeling, srt treatment group (*n* = 10, srt 8w) and the 16-week-old normal group (*n* = 10, sham 8w). All mice were purchased from Beijing Weitong Lihua Laboratory Animal Technology Co., Ltd. (Beijing, China). The breeding environment of mice is 22 °C, 60 ± 5% humidity environment, which can move freely and obtain food, and provide 12 h of light and dark cycle. The surgical site was the mouse's right knee joint. All operations were destabilizing the medial meniscus(DMM)operations [13]. SRT treatment group mice were injected intraperitoneally with SRT-1720 drug.

### SRT-1720 drug treatment

Drug SRT-1720 (a specific activator of SIRT1, APEX-bio) was dissolved in dimethyl sulfoxide (DMSO) at 38 mg/ml, and was stored at -20 °C. SRT-1720 was first diluted in phosphate-buffered saline (PBS), and was administered intraperitoneally twice a week at a dose of 2.5 mg/100 g. The drug treatment was started from day 1^st^ after the operation, and later on, the mice were sacrificed after 2 or 8 weeks of treatment using CO_2_ anesthesia. The fresh specimens of the right knee joint were obtained. The drug dosage and configuration of SRT-1720 were adopted from previous study [14].

### Histological evaluation of articular cartilage

Fresh human tibial plateau specimens and mouse knee specimens were collected for histological evaluation, 6 mice were randomly selected from each group. Knee specimens were immediately placed in 4% paraformaldehyde solution and fixed for 48 h. The specimen was rinsed with PBS (pH = 7.3), and the liquid was changed every hour for 6 h. When decalcified, 10% EDTA solution was added and replaced once daily for 3 weeks. Finally, the specimen was embedded in paraffin. Before observation, the specimens were cut into sagittal sections of 5 um. The specimens (human tibial plateau injury specimens and mice) were evaluated with saffine O solid green staining hematoxylin–eosin staining according to the OARSI grading criteria established by the International Association for Osteoarthritis Research [15]. The human samples were divided into OA mild group (grade 0–2), OA moderate group (grade 3–4), and OA severe group (grade 5–6).

### Immunohistochemistry and immunofluorescence

For immunohistochemical experiments, paraffin sections were first dewaxed and dehydrated in xylene using 0.1% trypsin to repair the antigen at 37 °C for 15 min and was then incubated in 3% hydrogen peroxide solution for 15 min to inhibit peroxidase activity. The sections were blocked with Goat serum for 30 min and then incubated with the primary antibody overnight at 4 °C. Primary antibodies include: Anti-SIRT1: (Abcam, 1:200, ab189494), Anti-β-catenin:(Proteintech,1:200,51,067–1-AP), Anti-LEF1:(proteintech,1:200,14,972–1-AP), Anti-MMP13:(abcam,1:300,ab39012), and Anti-collagen II: (abcam,1:300,ab34712). The number of positive cells was counted with Image pro-plus 6.0 software. The sections were fixed in 4% PFA for immunofluorescence experiments and then combined with the corresponding antibody at 4 °C overnight, followed by a combination with Alexa Fluor 488 goat anti-rabbit secondary antibody (Abcam, 1:300, ab150077) and incubated at 37 °C for 60 min.

An inverted fluorescence microscope was used to observe the positive cells, and Imagepro-plus 6.0 software was used to count the proportion of positive cells.

### RNA extraction and quantitative RT-qPCR

The Quantitative Real-time PCR(RT-qPCR) reaction analysis was performed according to the information specified in the real-time quantitative PCR (MIQE) guidelines issued in 2009. The total RNA was extracted using the tissue RNA extraction kit (AXYGEN, Wujiang, China). Each group comprised of 4 mouse knee cartilage. Total RNA (500 ng) was used to prepare cDNA (Bio-rad, Hercules, CA, USA). 25 ng cDNA and SYBER Green mixture (TransgenBiotech, Beijing, China) were used for the real-time quantitative PCR reaction. The primer sequence used are as follow:

**Table:** 

Gene	F-Primer	R-Primer
β-actin	5’-GTGCTATGTTGCTCTAGACTTCG-3’	5’-ATGCCACAGGATTCCATACC-3’
LEF-1	5’-GGCGGCGTTGGACAGATCAC-3’	5’-GGATGAGGGATGCCAGTTGTGTG-3’
SIRT-1	5’-AGTAACAGTGACAGTGGCACATGC-3’	5’-GCCTCTCCGTATCATCTTCCAAGC-3’

β-actin was used as a reference housekeeping gene. Relative expression of the target gene was determined using 2^−△△CT^ equation.

### Statistical analysis

One-way analysis of variance (One-Way ANOVA) method was used to compare multiple groups of data, and Tukey’s multiple comparisons test was used for homogeneity of variance. All the data were expressed as mean ± SD. *P* < 0.05 was considered statistically significant. GraphPad Prism 8 software for statistical analysis.

## Results

### Pathological staining of human knee cartilage and immunohistochemical detection of SIRT-1 protein

**Fig. 1:**
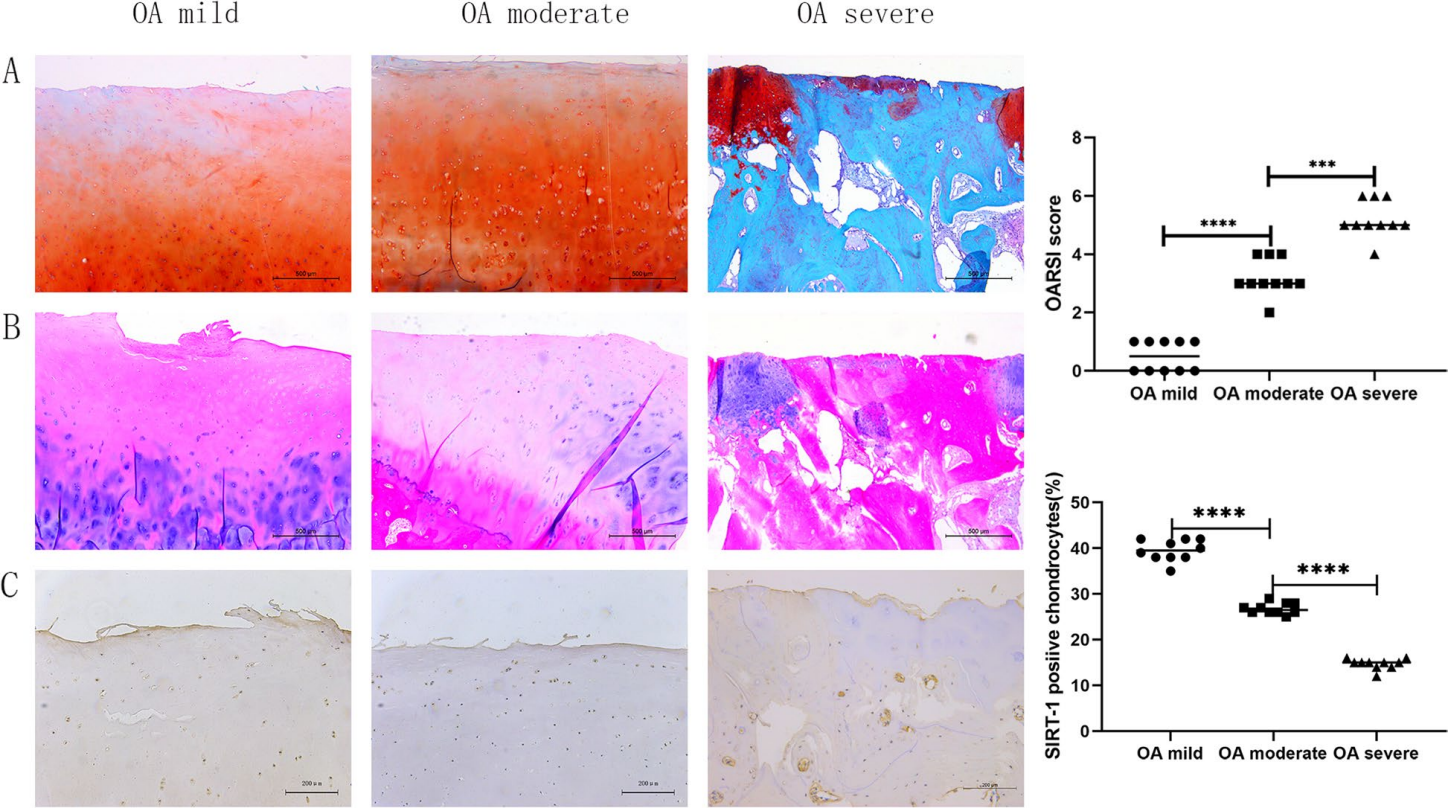
**A** Staining results of safranin O fast green in human tibial plateau specimens, 50x, scale bar = 500 μm; **B** Hematoxylin–Eosin staining experiment results, 50x, scale bar = 500 μm; **C** SIRT-1 immunohistochemistry test results, 100x, scale bar = 200 μm, (^****^P < 0.0001)

We used Safranin O Fast Green (Fig. 1A) and Hematoxylin–Eosin Staining (Fig. 1B) to evaluate the injured human cartilage specimens. Among them, the Safranin O Fast Green staining of cartilage was red, the hyaline cartilage was slightly stained, the subchondral bone was blue, and the nucleus of the chondrocyte was blue-brown. In HE staining, the cytoplasm and extracellular matrix were stained red, and the nucleus of the chondrocyte was purplish-blue. In the OA mild group, the surface of cartilage was smooth and continuous without cracks, and the chondrocytes were normal in number and shape. In OA moderate group, chondrocytes were reduced, the cells on the surface of cartilage are small, the deep was hypertrophic, and the nucleus was missing. In OA severe group, cartilage wear was severe, subchondral bone was exposed, and cartilage fissures were formed.

The SIRT-1 protein expression was detected by immunohistochemistry (Fig. 1C). The nucleus of positive cells is brown. The expression level of SIRT-1 protein was: OA mild > OA moderate > OA severe, and the expression level of SIRT-1 was inversely proportional to the severity of OA (*P* < 0.001). The differences in the relative expression level of SIRT-1 protein were Statistically significant.

### Pathological staining of the mouse knee joint and immunohistochemical detection of SIRT-1 and LEF-1 proteins

**Fig. 2:**
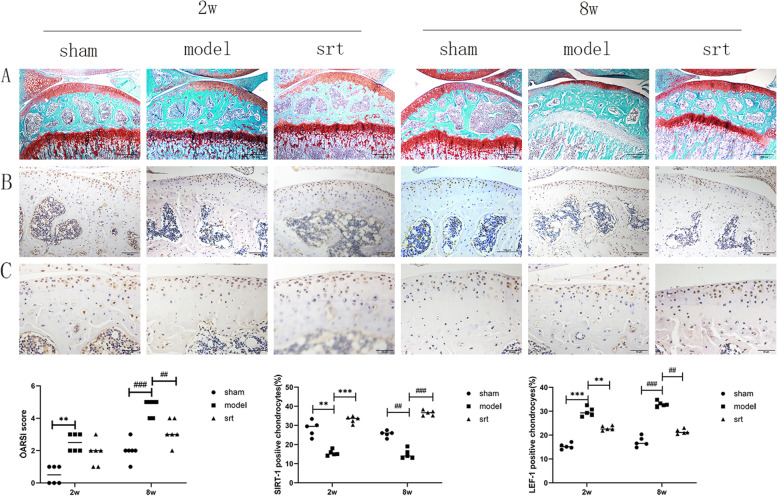
**A** Mouse Safranin O Fast Green Staining Results, 100x, scale bar = 200 μm; **B** SIRT-1 immunohistochemistry test results, 400x, scale bar = 50 μm; **C** LEF-1 immunohistochemistry test results, 400x, scale bar = 50 μm, (^***^P < 0.001; ^##^*P* < 0.01)

We stained animal tissue sections with Safranin O Fast Green and assessed articular cartilage degeneration using OARSI score (Fig. 2A). The mice’s articular surface in the 2-week and 8-week sham group was complete, smooth, and continuous, and the cartilage thickness was moderate. Among them, the thickness of the cartilage in the 8-week sham group became slightly thinner. The cartilage surface of the 2-week and 8-week model mice was discontinuous, and the number decreased. The 8-week model mice lost hyaline cartilage and exposed calcified cartilage. The OARSI score and histological analysis showed that compared with the normal group, the scores of model mice at 2-week and 8-week increased significantly, and the cartilage surface was severely damaged. Compared with the model mice, the cartilage destruction of the mice in the 2-week and 8-week SRT treatment group were fewer, and their OARSI scores were also reduced.

We used immunohistochemical techniques to detect SIRT-1 (Fig. 2B) and LEF-1 proteins (Fig. 2C). Compared with the 2-week and 8-week sham groups, the SIRT-1 expression was significantly decreased in the 2-week(*P* < 0.001) and 8-week SRT groups, whereas the expression of LEF-1 protein, was significantly increased. The expressions of the two proteins in the 2-week and 8-week model groups were between the sham and srt groups. These findings indicate that SIRT-1 and LEF-1 expression are inversely proportional when OA occurs, and SRT-1720 does activate the expression of SIRT-1.

### Immunohistochemical detection of osteoarthritis-related proteins

**Fig. 3:**
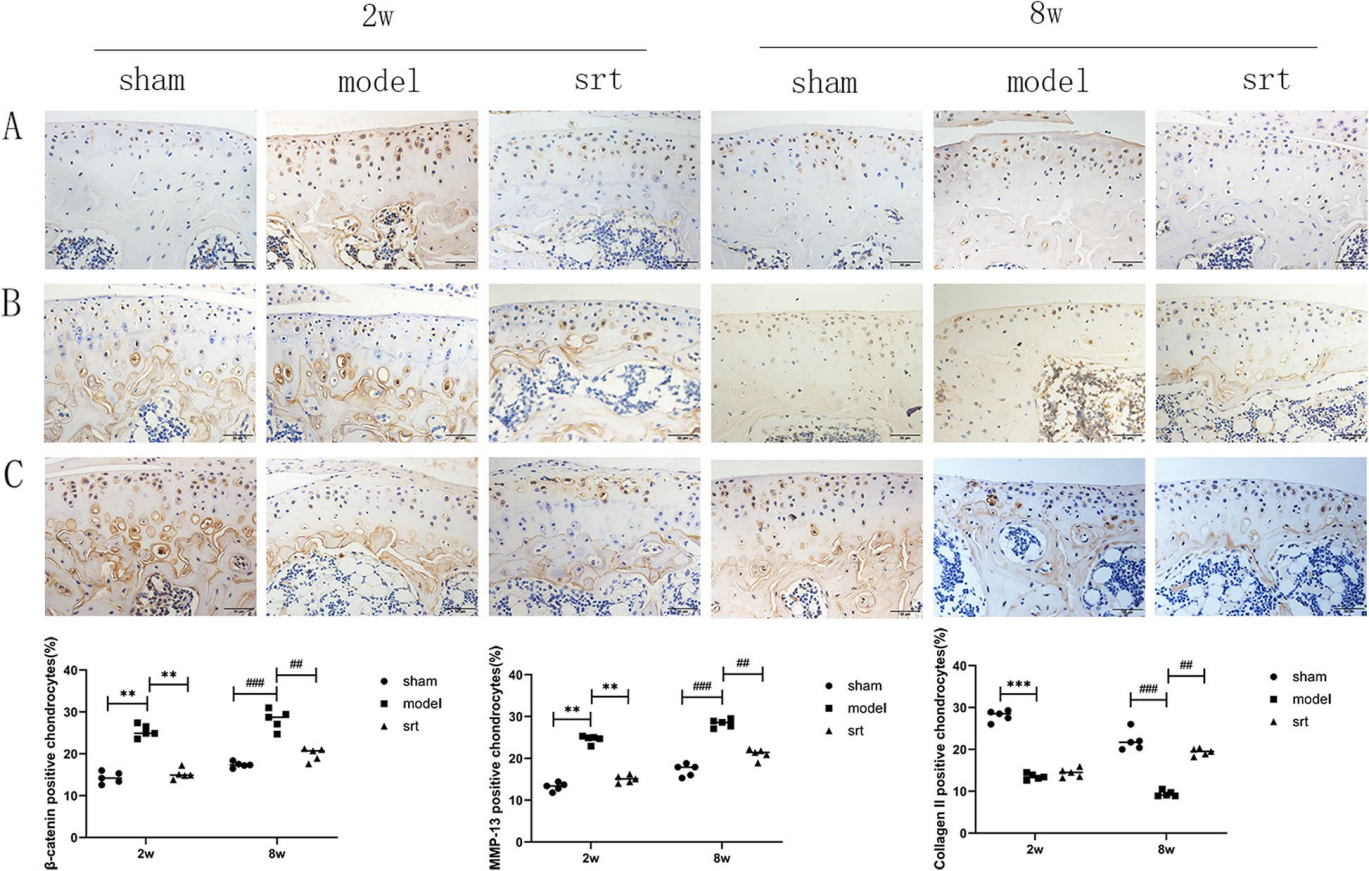
**A** β-catenin immunohistochemistry test results, 400 × , scale bar = 50 μm; **B** MMP-13: immunohistochemistry test results, 400 × , scale bar = 50 μm; **C** Collagen II:immunohistochemistry test results, 400 × , scale bar = 50 μm (^***^P < 0.001,^##^*P* < 0.01)

In this study, immunohistochemical experiments were conducted to investigate the changes of β-catenin (Fig. 3A), MMP-13 (Fig. 3B) and collagen II (Fig. 3C) and their chondroprotective effects during OA. β-catenin is a key upstream factor in the Wnt signaling pathway [16]. MMP-13 is the main degradation enzyme of collagen II in cartilage [17]. As a biomarker of OA, collagen II has a protective effect on OA [18]. Compared with the 2-week and 8-week sham group, the expressions of β-catenin and MMP-13 in the 2-week and 8-week model groups were increased, the Collagen II protein expression was reduced, the cartilage layer became thinner, and the cartilage cells were significantly reduced. Compared with the model group, the expressions of β-catenin and MMP-13, hypertrophy, and death of chondrocytes in the 2-week SRT treatment group and the 8-week SRT treatment group were reduced, and the cartilage layer was thickened.

### Immunofluorescence detection of osteoarthritis-related proteins

**Fig. 4:**
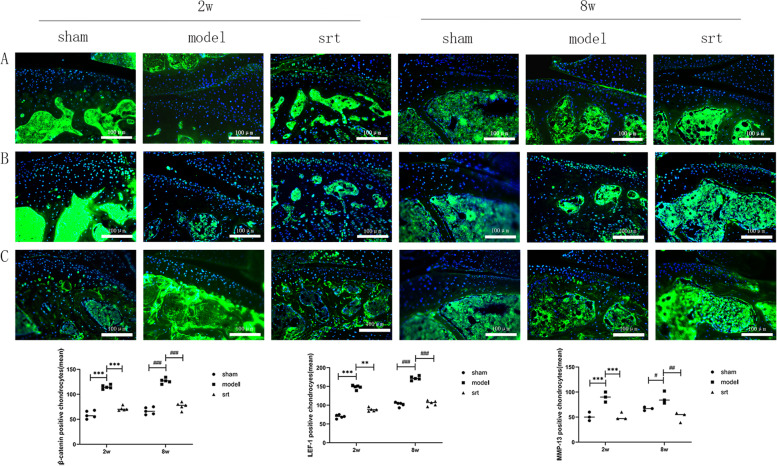
**A** β-catenin: Immunofluorescence experiment results, 200 × , scale bar = 100 μm; **B** LEF-1: Immunofluorescence experiment results, 200 × , scale bar = 100 μm; **C** MMP-13: Immunofluorescence experiment results, 200 × , scale bar = 100 μm ((^***^P < 0.001); ^##^*P* < 0.01))

We performed immunofluorescence experiments to detect the expression of β-catenin (Fig. 4A), LEF-1 (Fig. 4B) and MMP-13 proteins (Fig. 4C). Compared with the 2-week normal group and the 8-week normal group, the cartilage β-catenin, LEF-1, and MMP-13 protein expressions in the 2-week and 8-week models were increased, the cartilage layer became thinner and degenerated, and chondrocytes became hypertrophic and necrotic, and the extracellular matrix is reduced. Compared with the model, the expression of β-catenin, LEF-1, and MMP-13 in cartilage in the 2-week SRT treatment group and 8-week SRT treatment group decreased, the cartilage layer was thickened, the number of cartilage cells increased, and the extracellular matrix increased.

### Changes in mRNA expression of SIRT-1 and LEF-1 in osteoarthritis

**Fig. 5:**
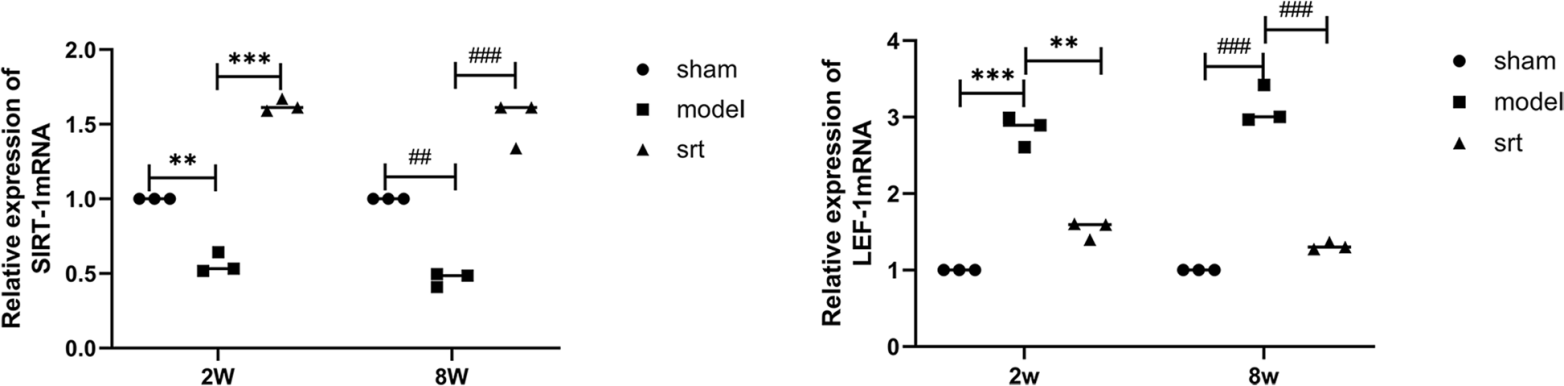
**A** The relative expression of SIRT-1 mRNA; **B** The relative expression of LEF-1 mRNA. (^***^P < 0.001, ^##^*P* < 0.01)

The real-time quantitative polymerase chain reaction (RT-qPCR) method was used to study the relative expression of SIRT-1 and LEF-1 mRNA during OA (Fig. 5). The results showed that compared with the sham group, the mRNA expression of SIRT-1 in the model group (2-week and 8-week) decreased sharply, while the mRNA expression of LEF-1 increased rapidly. Compared with the model group, the mRNA expression level of SIRT-1 increased in the SRT treatment group (2-week and 8-week) (*P* < 0.001).

## Discussion

This study shows that during the development of OA, the cartilage surface is gradually destroyed and damaged, cartilage cells became hypertrophic and necrotic, and the thickness of hyaline cartilage is gradually worn and thinned. On the contrary, the thickness of calcified cartilage gradually increases, and the cartilage surface of the severe group specimens is defective. The subchondral bone was exposed, hyaline cartilage disappeared in some specimens, and the OARSI rating increased. This clinically verifies the cartilage wear and joint pain that occurs in patients with OA. In this study, according to the OARSI grading, the corresponding pathological stained sections were divided into OA mild group, OA moderate group and OA severe group. The expression levels of different proteins measured in various groups are different.

The immunohistochemistry result showed that the expression of SIRT-1 protein gradually decreases with the increase of cartilage destruction, suggesting that its expression changes may be related to the occurrence and development of OA. In addition, when the expression of SIRT-1 protein decreasing, the expression trend of LEF-1 and β-catenin protein is opposite, and the expression level gradually increases. These are consistent with the results of the previous study [12], which were also confirmed by our qPCR experimental results, suggesting that SIRT-1 may have the regulatory effect of LEF-1. Type II collagen is the main collagen in the extracellular matrix of chondrocytes, which plays the role of nourishment, and protects chondrocytes [18]. Collagen II is the main collagen component in the extracellular matrix of chondrocytes and is closely related to the occurrence and progression of OA [18]. In this experiment, we found that with the increase of cartilage destruction, the expression of collagen II protein gradually decreases. This also confirms that type II collagen is closely related to cartilage destruction and loss.

Lymphocyte enhancer factor (LEF-1) exists in the nucleus of mammals. It can combine with β-catenin and DNA to form a ternary compound, regulating the bending and transcription of the corresponding DNA [19]. In animal experiments, we observed that when the severity of OA increased, the expression of SIRT-1 protein gradually decreased while the expression of LEF-1 increased, and cartilage thickness decreased simultaneously. However, after activating the SIRT-1 expression, the results were opposite, the cartilage layer thickened. It is confirmed that LEF-1 may achieve the chondroprotective effect of SIRT-1. Previous studies have shown that β-catenin and HDAC1 act in a LEF-1-dependent manner; compared with unbound HDAC1, the combined activity of HDAC1 is reduced [20]. In LEF-1-deficient mice, we observed that the skin, teeth, hair, etc., are missing, and their life span is significantly affected, suggesting that LEF-1 plays an important role in the growth and development of organisms [21].

It has been confirmed that SIRT-1 protein can regulate the expression of LEF-1 to play a chondroprotective effect in chondrocytes of patients with OA [12], and our results further verify this view through animal experiments. In this study, we used medial meniscus instability surgery (DMM) to induce OA in mice. This surgery is widely used to study OA in mice, and it can simulate the human OA development process. Another method of modeling-anterior cruciate ligament transection (ACLT) has also been reported that the development of OA is too fast, so this method is not used [22].In the pathological staining results, we observed that the degree of cartilage destruction in the 2-week and 8-week was significantly different, but the changes of related inflammatory proteins were not significant in the molecular biological experiments. This may be because the extracellular matrix of cartilage is rich in type II and type X collagen, proteoglycan and carbonated hydroxyapatite, etc. [23]; when OA occurs, these components interact with each other, the mechanism of action is complex, and the corresponding results appear.

As a small molecule activator, SRT-1720 can specifically increase the expression level of SIRT-1, regulate the energy metabolism efficiency of body tissues, and prevent metabolic disorders [14]. It has been reported to delay the progression of OA. In this study, we used this drug as the activator of SIRT-1 for related research. SIRT-1 is expressed in the nucleus of all cartilage and synovial tissues, and its deacetylation ability is related to the occurrence of various inflammations and diseases [24]. SIRT-1 can regulate the body's metabolic function, regulate the body's insulin sensitivity, and control inflammation [25]. In the cartilage of mice lacking the SIRT-1 gene, it was found that chondrocyte apoptosis increased, and the expression of collagen II in the extracellular matrix was significantly reduced [26]. On the contrary, the expression of aggregated metal matrix protease 13 (MMP-13) was significantly increased. Our immunohistochemistry results also verify these points. SIRT-1 can activate autophagy in human chondrocytes and exert chondroprotective function [27]. In addition, SIRT-1 is also involved in the pathogenesis of diseases such as acute kidney injury, craniocerebral trauma, and aortic sclerosis [28]. In these diseases, the SIRT-1 expression is reduced, while in some cancers, the SIRT-1 expression is significantly increased, suggesting that it may be used for cancer treatment [29]. Vorinostat (SAHA), as a natural HDAC inhibitor, has been shown to inhibit IL-1β-induced MMP-13 expression [30]. Furthermore, as a natural HDAC inhibitor, trichostatin can increase the ratio of matrix metalloproteinase inhibitor (TIMP-1)/MMP in OA mice to play a chondroprotective effect [31]. These studies show that HDAC inhibitors play a protective role in OA, which may be a new exciting research direction.

## Conclusion

SRT-1720, as a specific activator of SIRT-1, does increase the protein level of SIRT-1. SIRT-1 may play a protective role in cartilage by regulating the expression of LEF-1 and related inflammatory factors in OA.

## Data Availability

The datasets generated and/or analysed during the current study are not publicly available due to limitations of ethical approval involving the patient data and anonymity but are available from the corresponding author on reasonable request.

## Abbreviations

SIRT-1: Silent information regulator 1
LEF-1: Lymphocyte enhancer factor 1
MMP-13: Matrix metalloproteinase 13
OA: Osteoarthritis
ADAMTS: A Disintegrin And Metalloproteinase With Thrombospondin
HDAC: Histone deacetylase
TCF: T-cell factor
HMG: High mobility group
DMM: Destabilizing the medial meniscus
DMSO: Dimethyl sulfoxide
PBS: Phosphate-buffered saline
OARSI: Osteoarthritis Research Society International
RT-qPCR: Quantitative Real-time PCR
LRP5/6: Low-density lipoprotein receptor-related protein 5/6
ACLT: Anterior cruciate ligament transection
NAD^+^: Nicotinamide adenine dinucleotide
TSA: Trichostatin
TIMP1: Metallopeptidase inhibitor 1
